# Schistosomiasis, Soil-Transmitted Helminthiasis, and Sociodemographic Factors Influence Quality of Life of Adults in Côte d'Ivoire

**DOI:** 10.1371/journal.pntd.0001855

**Published:** 2012-10-04

**Authors:** Thomas Fürst, Kigbafori D. Silué, Mamadou Ouattara, Dje N. N'Goran, Lukas G. Adiossan, Yao N'Guessan, Fabian Zouzou, Siaka Koné, Eliézer K. N'Goran, Jürg Utzinger

**Affiliations:** 1 Department of Epidemiology and Public Health, Swiss Tropical and Public Health Institute, Basel, Switzerland; 2 University of Basel, Basel, Switzerland; 3 Unité de Formation et de Recherche Biosciences, Université de Cocody, Abidjan, Côte d'Ivoire; 4 Centre Suisse de Recherches Scientifiques en Côte d'Ivoire, Abidjan, Côte d'Ivoire; 5 Programme National de Lutte contre la Schistosomiase, les Géohelminthiases et la Filariose Lymphatique, Abidjan, Côte d'Ivoire; 6 Hôpital Général de Taabo, Taabo Cité, Côte d'Ivoire; Universidade Federal de Minas Gerais, Brazil

## Abstract

**Background:**

Burden of disease estimates are widely used for priority setting in public health and disability-adjusted life years are a powerful “currency” nowadays. However, disability weights, which capture the disability incurred by a typical patient of a certain condition, are fundamental to such burden calculation and their determination remains a widely debated issue.

**Methodology:**

A cross-sectional epidemiological survey was conducted in the recently established Taabo health demographic surveillance system (HDSS) in south-central Côte d'Ivoire, to provide new, population-based evidence on the disability caused by schistosomiasis and soil-transmitted helminthiasis. Parasitological results from stool, urine, and blood examinations were juxtaposed to quality of life (QoL) questionnaire results from 187 adults. A multivariable linear regression model with stepwise backward elimination was used to identify significant associations, considering also sociodemographic characteristics obtained from the Taabo HDSS database.

**Principal Findings:**

Prevalences for hookworm, *Plasmodium* spp., *Trichuris trichiura*, *Schistosoma haematobium* and *Schistosoma mansoni* were 39.0%, 18.2%, 2.7%, 2.1% and 2.1%, respectively. *S. mansoni* and *T. trichiura* infections of any intensity reduced the participants' self-rated QoL by 16 points (95% confidence interval (CI): 4–29 points) and 13 points (95% CI: 1–24 points), respectively, on a scale from 0 (worst QoL) to 100 points (best QoL). The only other statistically significant effect was a 1-point (95% CI: 0.1–2 points) increase on the QoL scale per one unit increase in a calculated wealth index.

**Conclusions/Significance:**

We found consistent and significant results on the negative effects of schistosomiasis and soil-transmitted helminthiasis on adults' self-rated QoL, also when taking sociodemographic characteristics into account. Our results warrant further investigation on the disability incurred by helmintic infections and the usefulness of generic QoL questionnaires in this endeavor.

## Author Summary

In public health, "burden" estimates should capture the human suffering caused by certain health states, and the estimates are often used for priority setting. However, such "burden" estimates need to assess not only the number of affected people by certain conditions, but also the disability incurred by the average patient, and the determination of the degree of disability remains a widely debated issue. In order to provide new, population-based evidence on the disability caused by infections with parasitic worms, we administered a quality of life (QoL) questionnaire to 187 adults in rural Côte d'Ivoire and concurrently examined them for parasitic worm infections. We also considered sociodemographic characteristics in our analysis. In comparison with their non-infected counterparts, infected people reported a 13–16 points lower QoL on a scale from 0 (worst QoL) to 100 points (best QoL). At the same time, a one unit increase in a calculated wealth index revealed a 1-point increase in the participants' QoL. The results are consistent and warrant further investigation on the disability induced by parasitic worm infections and the usefulness of QoL questionnaires in this endeavor.

## Introduction

Efforts are underway for a comprehensive revision of the global burden due to major diseases, injuries, and risk factors [1]. The initial global burden of diseases, injuries, and risk factors study, commissioned by the World Bank more than 20 years ago, introduced the disability-adjusted life year (DALY) metrics [2]. DALY is a time-based measure, which combines years of life lost (YLL) due to premature death, and years of life lived with disability (YLD) due to a certain condition [2]. Results from the initial global burden of disease study have been widely used for priority setting in research, policy, and practice, and the DALY became a powerful “currency” in public health (see for example reference [3]).

An undeniable merit of the global burden of disease concept is the renewed interest in descriptive epidemiology and population health measurement. Not surprisingly though, the concept also stimulated considerable controversies. Amongst other issues, criticism about the disability weights, which should measure the disability caused by a certain condition on a continuous scale from 0 (perfect health) to 1 (death), was raised as the original disability weights were solely based on expert opinion [2], [4], [5]. In order to respond to such shortcomings, the disability weights will be adapted in the current revisions of the global burden of diseases, injuries, and risk factors. According to the operations manual on the project's homepage [6], the revised disability weights will be elicited not only by expert opinion, but also by an Internet-supported, multi-method study among qualified respondents and, for selected sequelae, by population-based discrete choice assessments.

In order to provide new, setting-specific, population-based evidence on the disability caused by schistosomiasis and soil-transmitted helminth infections, we conducted a cross-sectional survey among adults in the Taabo health demographic surveillance system (HDSS), in south-central Côte d'Ivoire. Generic quality of life (QoL) questionnaires and standardized, quality-controlled parasitological methods were applied and the results juxtaposed, taking into consideration readily available sociodemographic data from the Taabo HDSS database as potential confounders. To our knowledge, only few studies employed generic QoL questionnaires to assess disability attributable to helminth infections [7]–[10]. However, these studies focused on school-aged children [8], [9] and other narrowly defined population subgroups (e.g., patients with advanced, chronic infections) [7], [10]. The present study provides new insight regarding the usefulness and applicability of generic QoL questionnaires to elicit disabilities due to helminthiases in the general public.

## Materials and Methods

### Ethics Statement

The study protocol was approved by the institutional research commissions of the Swiss Tropical and Public Health Institute (Swiss TPH; Basel, Switzerland) and the Centre Suisse de Recherches Scientifiques en Côte d'Ivoire (CSRS; Abidjan, Côte d'Ivoire). Ethical clearance was provided by the ethics committee of Basel (EKBB; reference no. 316/08) and the Comité National d'Ethique et de la Recherche (CNER) in Côte d'Ivoire (reference no. 1086 MSHP/CNER).

The Taabo HDSS was set-up in mid-2008, located in the Taabo area in the south-central part of Côte d'Ivoire. While establishing the Taabo HDSS, district and village authorities and the general public were informed about its purpose, operational procedures, potential risks, and benefits. The present study was carried out in June 2010, readily embedded in the second cross-sectional epidemiological survey pursued once every year. Written informed consent was obtained from all participants. It was emphasized that participation was voluntary, and hence people could withdraw anytime without further obligation. All results were coded and kept confidential. At the end of the study, all people living in the Taabo HDSS were invited for deworming with albendazole (400 mg single oral dose) and ivermectin (∼200 µg/kg using a dose pole) irrespective of participants' infection status [11], [12]. Additionally, a physician was present during our study and treated acute cases of infections, other diseases and injuries, or referred people to the district hospital in Taabo Cité if need be. Preventive chemotherapy against schistosomiasis, using praziquantel (∼40 mg/kg according to a dose pole), was administered 6 months later.

### Study Area and Population

**Figure 1:**
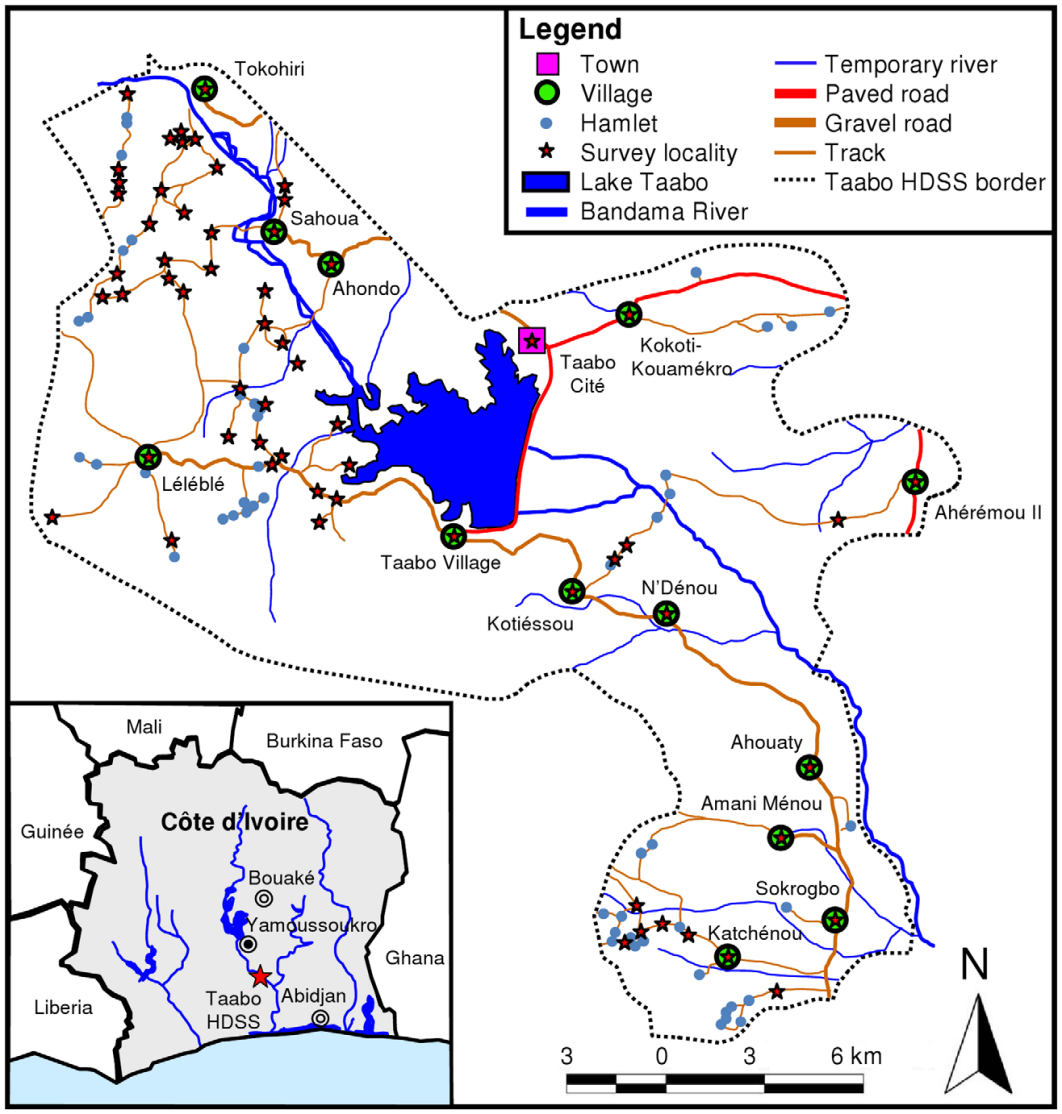
Map of the Taabo health demographic surveillance system (HDSS) and predefined survey locations. The study was carried out in June 2010, readily embedded in the second annual cross-sectional epidemiological survey of the Taabo HDSS.

The Taabo HDSS covers most of the rural Sous-Préfecture Taabo. Its main office is located in Taabo Cité, 160 km northwest of Abidjan. The region's tropical climate follows a seasonal pattern with a long dry season between November and April and two rainy seasons, a long one between April and July and a shorter one in September and October [13], [14]. The area is at the interface of tropical rainforest in the south and the savannah in the north with the Bandama River running through from north to south. In 1979, construction of a large hydroelectric dam was completed (Figure 1) [15]. Malaria [16] and neglected tropical diseases (e.g., schistosomiasis [13], [15], [17] and soil-transmitted helminthiasis [13], [18]) are endemic and rapid re-infection of *Schistosoma haematobium* has been observed after praziquantel administration [17].

Vital statistics (i.e., pregnancy, birth, death, in-migration, and out-migration) and the health of some 38,500 individuals registered in the Taabo HDSS are monitored longitudinally. People are mainly engaged in subsistence farming of manioc, yams, and banana, while cacao and coffee are farmed as cash crops [13], [14]. There are also some fishermen around Lake Taabo, some artisans, and – particularly in Taabo Cité – some shopkeepers and businessmen.

### Individual and Household Data Compilation

For the present study, sociodemographic data on individual and household level were obtained from the Taabo HDSS database. Individual data included sex, age, relationship with the head of household, education, and main occupation. Household-level data contained information on the households' location, the number of household members, housing construction material, availability of certain facilities, and the possession of equipment.

### Field and Laboratory Procedures

All collaborators, including local health personnel, were trained and informed about the purpose, procedures, potential risks, and benefits of the cross-sectional survey and the deworming. Subsequently, key informants, field enumerators, and supervisors of the Taabo HDSS and the local health personnel informed all heads of households to visit, together with their families, a predefined, nearby survey location on a specified date and time to receive anthelmintic treatment. In addition, approximately 7% of all households in the Taabo HDSS were selected by stratified random sampling. These households were visited the day before the treatment and two plastic containers were distributed to all household members for collection of a lemon-sized fresh morning stool and a urine sample the next day.

**Figure 2:**
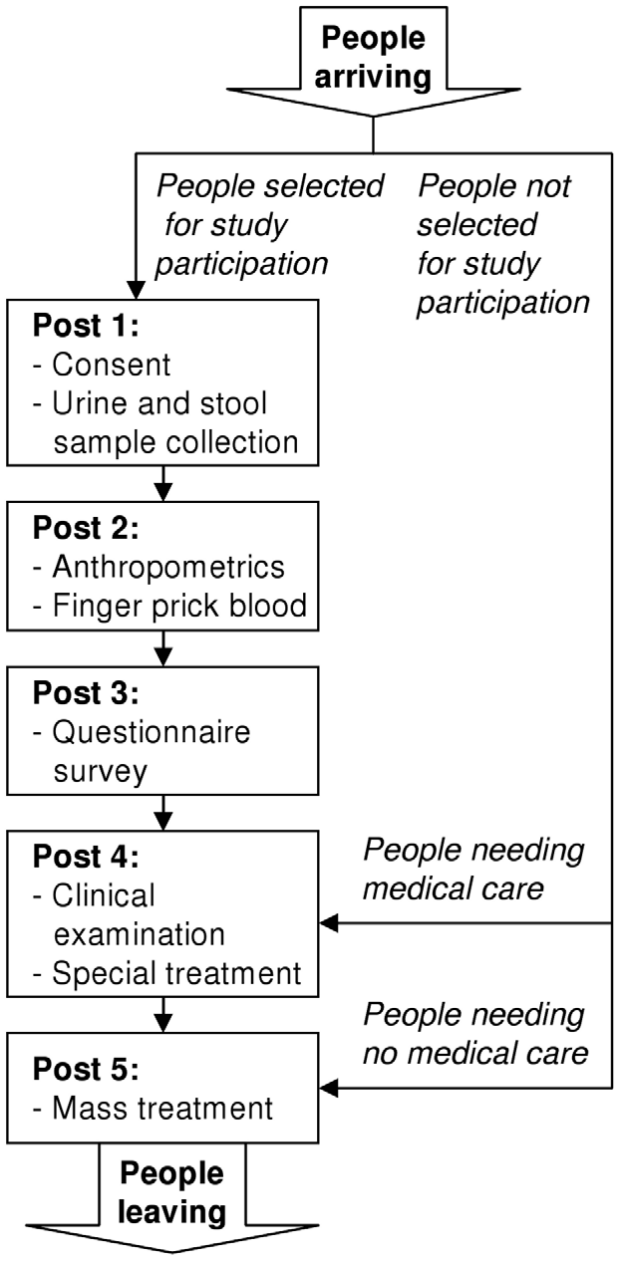
Flow chart of the study procedure in the field. The study was carried out in June 2010, readily embedded in the second annual cross-sectional epidemiological survey of the Taabo health demographic surveillance system (HDSS). In the frame of this second annual cross-sectional epidemiological survey, the whole population of the Taabo HDSS was offered anthelmintic treatment with albendazole and ivermectin. At the same time, people selected for an in-depth clinical and parasitological examination were invited to visit a series of different posts, including a quality of life (QoL) questionnaire for heads of households and a second adult household member of the opposing sex.

On the day of treatment, people not selected for in-depth clinical and parasitological examinations received albendazole and ivermectin and they could continue with their daily chores (Figure 2). People who suffered clinical episodes (e.g., infants with an axillary temperature ≥37.5°C and a history of fever) were seen by a physician and provided antimalarial treatment or other specific interventions if need be. Families selected for clinical and parasitological examinations were sent to a first post to provide written informed consent and collection of stool and urine samples. At a second post, qualified technicians measured participants' weight and height and took a finger prick blood sample. One drop of blood was taken for a rapid diagnostic test (RDT) for malaria (ICT ML01 malaria Pf kit, ICT Diagnostics; Cape Town, South Africa), another drop for the analysis of hemoglobin (HemoCue Hb 301 System, HemoCue; Ängelholm, Sweden), and another two drops for thin and thick blood film preparation on microscope slides. At post three, trained field enumerators invited the heads of households and, if possible, a second adult household member of the opposite sex to independently fill out a questionnaire. At the next post, participants were clinically examined by a physician and, if need be, given specific treatment according to national guidelines. In a last step, participants were sent to the albendazole and ivermectin treatment post.

Blood, stool, and urine samples were transferred to the laboratory of the hospital in Taabo Cité and worked up the same day using standardized, quality-controlled techniques as described elsewhere [13], [14], [19]–[21]. In short, duplicate 41.7 mg Kato-Katz thick smears were prepared from each stool sample. After a clearing time of 30–45 min, the thick smears were examined under a microscope for soil-transmitted helminths (*Ascaris lumbricoides*, *Trichuris trichiura*, and hookworm) and *Schistosoma mansoni*. The sum of the helminth-specific egg counts of the two Kato-Katz thick smears were multiplied by a factor 12 to obtain infection intensities, as expressed in eggs per gram of stool (EPG). Urine samples were vigorously shaken, 10 ml drawn up into a syringe and pressed through a meshed nylon filter with a pore size of 20 µm (Sefar AG; Heiden, Switzerland). Next, filters were placed on a microscope slide and, after adding a drop of Lugol, examined for *S. haematobium* eggs under a light microscope. All parasitological examinations were performed by experienced laboratory technicians. For quality control, ∼5% of all microscope slides were re-examined by a senior technician. In case of disagreement, slides were read a third time and the result discussed among the technicians until agreement was reached. Thin and thick blood films were stained with Giemsa and examined for *Plasmodium* parasitemia.

### Questionnaire Survey

We used previously employed questionnaires in Côte d'Ivoire [22]–[25] and developed them further so that they allowed us to assess risk factors, signs, and symptoms related to neglected tropical diseases and malaria, and added one section pertaining to the respondent's QoL. The World Health Organization (WHO) Quality of Life-BREF (WHOQOL-BREF) questionnaire [26] served as template for the section on QoL, as previous studies with other generic questionnaire tools revealed ambiguous results in the People's Republic of China [7], [8], [10] and also in a first pre-testing in the frame of another study in the Taabo HDSS [13] (data on pre-testing of QoL questionnaires not shown).

The initial version of the questionnaire after translation into French was discussed with the field enumerators and supervisors of the Taabo HDSS. These field enumerators and supervisors are locals, who live in the different communities of the Taabo HDSS, are able to read, write, and speak French as well as the local languages Baoulé, Dioula, or Senufo. The questionnaire was further adapted based on their comments, then pre-tested in a nearby village and again refined in order to obtain the finally applied version (Text S1; for an unofficial translation into English see Text S2). During the survey, the same field enumerators and supervisors of the Taabo HDSS conducted the interviews, either in French or any of the aforementioned local languages.

### Statistical Analysis

Data were double-entered and cross-checked in EpiInfo version 3.5.1 (Centers for Disease Control and Prevention; Atlanta, United States of America) and analyzed in STATA version 10.1 (STATA Corp.; College Station, United States of America). For convenience, the myriad of main occupations obtained from the Taabo HDSS database were categorized into primary economic sector (i.e., making direct use of natural resources, such as farming), secondary economic sector (i.e., producing manufactured and other processed goods), and tertiary economic sector (i.e., producing services, such as education and health care), with housewives included in the primary sector as they are usually involved in (subsistence) farming. Socioeconomic household data were used to calculate an asset-based wealth index and deduce the inhabitants' socioeconomic status, according to an approach put forth in a World Bank publication [27]. The results on soil-transmitted helminth infections and schistosomiasis were classified into infection intensities (light, moderate, and heavy) according to WHO guidelines [11]. Malaria results from RDTs could only be considered as binary variables (positive/negative). Information on QoL was analyzed and summarized according to the WHOQOL user manual [28]. Questionnaire answers on QoL were coded as 1, 2, 3, or 4 with higher scores indicating elevated QoL (Text S1 and Text S2). The individual scores from questions 11, 12, 16, 17, 18, and 24 were summed up to form the score on domain 1 about the environmental wellbeing; the individual scores from questions 19, 21, 22, 23, and 25 were added and formed the score on domain 2 about the psychological wellbeing; the individual scores from questions 10, 13, 20, 26, 27, 28, and 29 were summed up to form the score on domain 3 about the physical wellbeing; and the individual scores from questions 14 and 15 were added and formed the score on domain 4 about the social wellbeing. All individual scores from questions 9 to 29 were summed up to form each participant's overall score on QoL. All scores were transformed to values between 0 and 100 (i.e., percentages) according to equation (1):
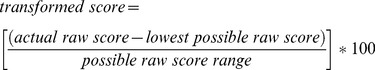
(1)


A Kruskal-Wallis test was performed to check for statistically significant (*p*<0.05) variations in the mean QoL scores assessed by the different interviewers (i.e., check for inter-observer variation). Furthermore, to assess the internal consistency and validity of the resulting QoL scores, we used Cronbach's alpha and a univariable linear regression model with the calculated overall QoL scores as outcome and the QoL ratings directly expressed by the participants in the final question of the questionnaire as explanatory variable (Text S1 and Text S2, see question 30: “How would you rate your quality of life in general? Very good? Good? Bad? Very bad?”).

Wilcoxon rank sum and Kruskal-Wallis test, χ^2^ and Fisher's exact test were employed, as appropriate, to check for statistically significant univariable associations between the different sociodemographic, parasitological, and Qol indicators. The outcome on QoL was further scrutinized in a multivariable linear regression analysis with sociodemographic data (i.e., age, sex, education, occupation, and socioeconomic status) and parasitological findings (i.e., schistosomiasis, soil-transmitted helminth infections, and malaria) as explanatory variables, considering also potential clustering of the results in interviewers and residential areas. A stepwise backward elimination procedure of non-significant explanatory factors was adopted to identify those variables most significantly influencing the participants' scores on QoL. In each iteration, the explanatory variable with the highest *p*-value was eliminated as long as the Akaike information criterion (AIC) was decreasing and the likelihood ratio test indicated no statistically significant association between the eliminated explanatory variable and the QoL scores. Categories of the same explanatory variable were combined, based on expert knowledge and logical deduction, before eventually eliminating the respective variable.

Only participants with signed written informed consent and complete data records (i.e., responses to all questions, duplicate Kato-Katz thick smears, urine filtration, and RDT for malaria) were included in the final analysis. Participants with completed written informed consent and questionnaire, but incomplete parasitological results were included in an attrition analysis.

## Results

### Operational Results and Sociodemographic Characteristics

**Figure 3:**
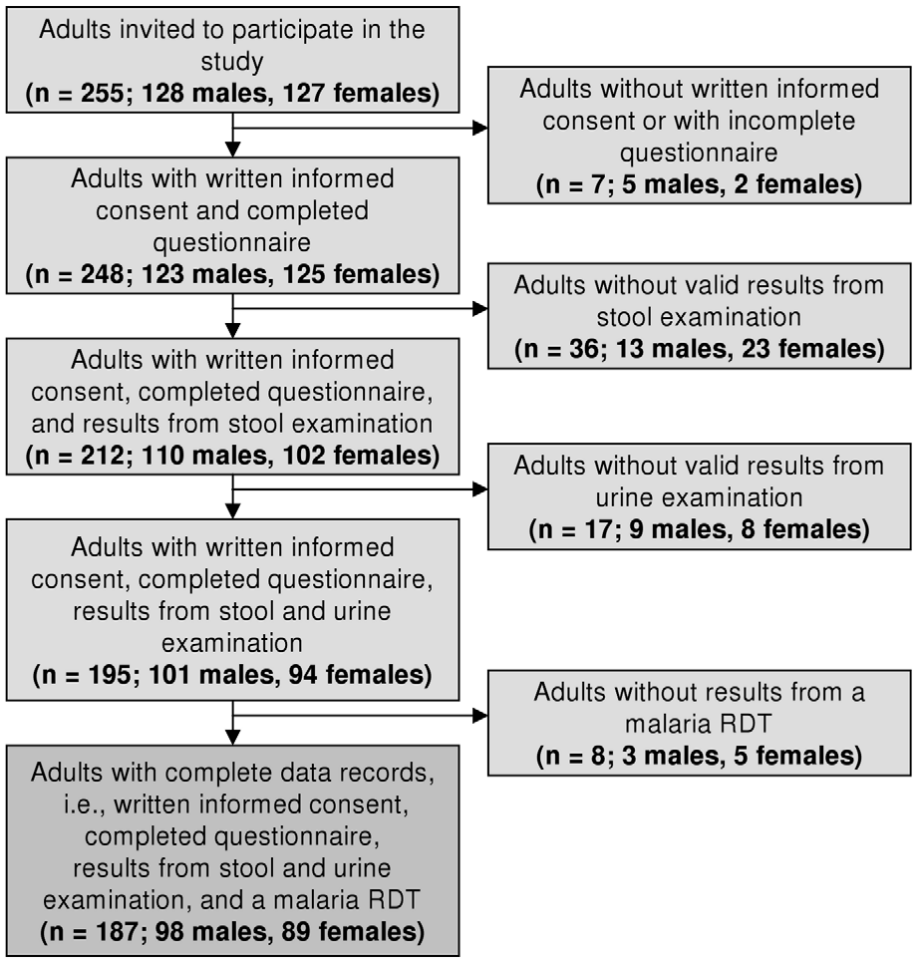
Flow chart of the participation and compliance in the present study. The study was carried out in June 2010, readily embedded in the second annual cross-sectional epidemiological survey of the Taabo health demographic surveillance system.

Overall, 255 adults were invited to participate in the study (Figure 3). Seven did not provide written informed consent or were unwilling to participate in the questionnaire survey, mostly because they were pressed for time. Moreover, 36 adults had no valid results from stool examination, as they failed to provide sufficiently large stool samples for duplicate Kato-Katz thick smear examination. Another 17 individuals had no valid results from the urine examination and eight were excluded as they did not have any malaria RDT results. Hence, the final study sample consisted of 187 adults; 98 males and 89 females. The median age was 45 years for both males (range: 18–87 years) and females (range: 21–83 years) (*p* = 0.926). About half (n = 92, 49.2%) of the participants were heads of household.

The educational level and the main sector of occupation are summarized in Table 1. The data on education reveal that a substantial number of participants have never attended school and, whereas the differences between age groups were not statistically significant (*p* = 0.663), women reported a significantly lower educational level than men (*p* = 0.046). Differences in occupational sector categorization showed no statistically significant difference between sex (*p* = 0.295) and age groups (*p* = 0.218) with most participants working in the primary sector (mainly subsistence farming) and only very few in the secondary sector. Not surprisingly, higher educated people were more often working in the tertiary rather than in the primary or secondary sectors (*p*<0.001).

**Table 1:** Educational level and main sector of occupation among 187 adults in rural Côte d'Ivoire.

Age	Sex	Number	Educational level				Main sector of occupation		
(years)		asked	None	Primary	Secondary	Higher	Primary^a^	Secondary^b^	Tertiary^c^
				school	school	education			
18–40	Male	37	15	11	9	2	31	1	5
	Female	29	19	5	4	1	23	0	6
41–60	Male	36	12	10	13	1	26	1	9
	Female	48	30	8	8	2	29	4	15
60+	Male	25	17	1	7	0	19	1	5
	Female	12	7	4	1	0	8	1	3
All	Male	98	44	22	29	3	76	3	19
All	Female	89	56	17	13	3	60	5	24
All	Both	187	100	39	42	6	136	8	43

Educational level and main sector of occupation among 187 adults interviewed in the Taabo health demographic surveillance system, south-central Côte d'Ivoire, in June 2010. Results are stratified by age and sex.

a Participants being farmer, fisher, hunter, or housewife.

b Participants being builder or artisan.

c Participants being driver, housekeeper, watchman, merchant, trader, hairdresser, gastronome, healer, nurse, teacher, student, office worker, or policeman.

Results from the socioeconomic analysis are shown in Table 2. According to the wealth quintiles, participants' socioeconomic status were not significantly related to sex (*p* = 0.377) or age group (*p* = 0.060), but positively associated with the educational level and working in the tertiary sector (both *p*<0.001).

**Table 2:** Overview of asset possession and the calculated socioeconomic status among 187 adults in rural Côte d'Ivoire.

Asset	Percentage of participants possessing the asset					
	Total	Wealth quintiles				
		Most	Very	Poor	Less	Least
		poor	poor	(n = 38)	poor	poor
		(n = 39)	(n = 36)		(n = 37)	(n = 37)
Type of housing						
Traditional hut	31.0	66.7	63.9	18.4	5.4	0.0
Barrack	1.1	5.1	0.0	0.0	0.0	0.0
Collective dwelling	1.1	0.0	0.0	0.0	2.7	2.7
Simple house	7.0	0.0	0.0	2.6	8.1	24.3
Row house	18.7	0.0	2.8	5.3	40.5	46.0
Modern house	22.5	0.0	11.1	42.1	32.4	27.0
Other housing	18.7	28.2	22.2	31.6	10.8	0.0
People per sleeping room^a^	2.1	2.3	1.9	1.8	2.5	2.2
Main lighting at home						
Lantern	29.4	87.2	58.3	0.0	0.0	0.0
Fix electric lighting	65.8	0.0	30.6	100.0	100.0	100.0
Other lighting	4.8	12.8	11.1	0.0	0.0	0.0
Energy source for cooking						
Wood	80.8	100.0	94.4	100.0	81.1	27.0
Wood+coal	10.7	0.0	0.0	0.0	13.5	40.5
Coal	3.7	0.0	0.0	0.0	5.4	13.5
Gas+coal	1.6	0.0	5.6	0.0	0.0	2.7
Gas	3.2	0.0	0.0	0.0	0.0	16.2
Equipment						
Hand barrow	9.6	0.0	11.1	5.3	13.5	18.9
Cistern	32.6	30.8	22.2	50.0	29.7	29.7
Mobile phone	67.4	20.5	80.6	65.8	78.4	94.6
Radio	64.2	46.2	69.4	55.3	64.9	86.5
TV	33.2	0.0	0.0	23.7	54.1	89.2
Pirogue	6.4	2.6	8.3	10.5	8.1	2.7
Bicycle	73.8	76.9	72.2	79.0	73.0	67.6
Moped	13.9	0.0	0.0	13.2	13.5	43.2
Ventilator	27.8	0.0	0.0	13.2	46.0	81.1
Fridge	5.9	0.0	0.0	0.0	2.7	27.0
Freezer	5.9	0.0	0.0	0.0	0.0	29.7

Overview of asset possession and the calculated socioeconomic status among 187 adults interviewed in the Taabo health demographic surveillance system, south-central Côte d'Ivoire, in June 2010.

a Reports the average number of people per sleeping room in the respective wealth quintile.

### Parasitological Results

Sex- and age-specific prevalence and intensity of helminth infection and *Plasmodium* infection are summarized in Table 3. We found hookworm, *Plasmodium* spp., *T. trichiura*, *S. haematobium* and *S. mansoni* prevalences of 39.0%, 18.2%, 2.7%, 2.1% and 2.1%, respectively. Most helminth infections were of light intensity and no heavy infections were diagnosed at all. With the exception of a higher prevalence of *Plasmodium* spp. infection in people aged 60 years and above (*p* = 0.028), no significant differences occurred in the prevalence and intensity of helminth and *Plasmodium* infection with regard to sex, age group, educational level, and occupational sector. Regarding participants' socioeconomic status, hookworm prevalence (*p*<0.001) and infection intensity (*p* = 0.002) as well as *T. trichiura* prevalence (*p* = 0.025) were significantly lower in wealthier participants.

**Table 3:** Prevalence and intensities of helminth and *Plasmodium* spp. infections, stratified by age and sex among 187 adults in rural Côte d'Ivoire.

Parasitic infection (in %)		18–40 years old		41–60 years old		60+ years old		All ages		
		Male	Female	Male	Female	Male	Female	Male	Female	Both sexes
		(n = 37)	(n = 29)	(n = 36)	(n = 48)	(n = 25)	(n = 12)	(n = 98)	(n = 89)	(n = 187)
*S. haematobium* a	Negative	100.0	100.0	100.0	93.7	96.0	100.0	99.0	96.6	97.9
	Light	0.0	0.0	0.0	6.3	4.0	0.0	1.0	3.4	2.1
	Heavy	0.0	0.0	0.0	0.0	0.0	0.0	0.0	0.0	0.0
*S. mansoni* b	Negative	97.3	100.0	100.0	93.7	100.0	100.0	99.0	96.6	97.9
	Light	2.7	0.0	0.0	4.2	0.0	0.0	1.0	2.3	1.6
	Moderate	0.0	0.0	0.0	2.1	0.0	0.0	0.0	1.1	0.5
	Heavy	0.0	0.0	0.0	0.0	0.0	0.0	0.0	0.0	0.0
Hookworm^b^	Negative	56.8	62.1	63.9	64.6	52.0	66.7	58.1	64.0	61.0
	Light	43.2	37.9	33.3	35.4	40.0	33.3	38.8	36.0	37.4
	Moderate	0.0	0.0	2.8	0.0	8.0	0.0	3.1	0.0	1.6
	Heavy	0.0	0.0	0.0	0.0	0.0	0.0	0.0	0.0	0.0
*A. lumbricoides* b	Negative	100.0	100.0	100.0	100.0	100.0	100.0	100.0	100.0	100.0
	Light	0.0	0.0	0.0	0.0	0.0	0.0	0.0	0.0	0.0
	Moderate	0.0	0.0	0.0	0.0	0.0	0.0	0.0	0.0	0.0
	Heavy	0.0	0.0	0.0	0.0	0.0	0.0	0.0	0.0	0.0
*T. trichiura* b	Negative	94.6	96.5	100.0	100.0	96.0	91.7	96.9	97.8	97.3
	Light	5.4	3.5	0.0	0.0	4.0	0.0	3.1	1.1	2.2
	Moderate	0.0	0.0	0.0	0.0	0.0	8.3	0.0	1.1	0.5
	Heavy	0.0	0.0	0.0	0.0	0.0	0.0	0.0	0.0	0.0
*Plasmodium* spp.^c^	Negative	78.4	79.3	88.9	89.6	68.0	75.0	79.6	84.3	81.8
	Positive	21.6	20.7	11.1	10.4	32.0	25.0	20.4	15.7	18.2

Prevalence and intensities of helminth and *Plasmodium* spp. infections among 187 adults examined in the Taabo health demographic surveillance system, south-central Côte d'Ivoire, in June 2010. The thresholds of helminth infection intensities are in accordance with WHO guidelines provided in reference [11].

a Prevalence obtained by urine filtration method (one urine sample per person, single filtration).

b Prevalence obtained by Kato-Katz method (one stool sample per person, duplicate Kato-Katz thick smears per sample).

c Prevalence obtained by rapid diagnostic test (one RDT per person).

### Self-Reported QoL

**Figure 4:**
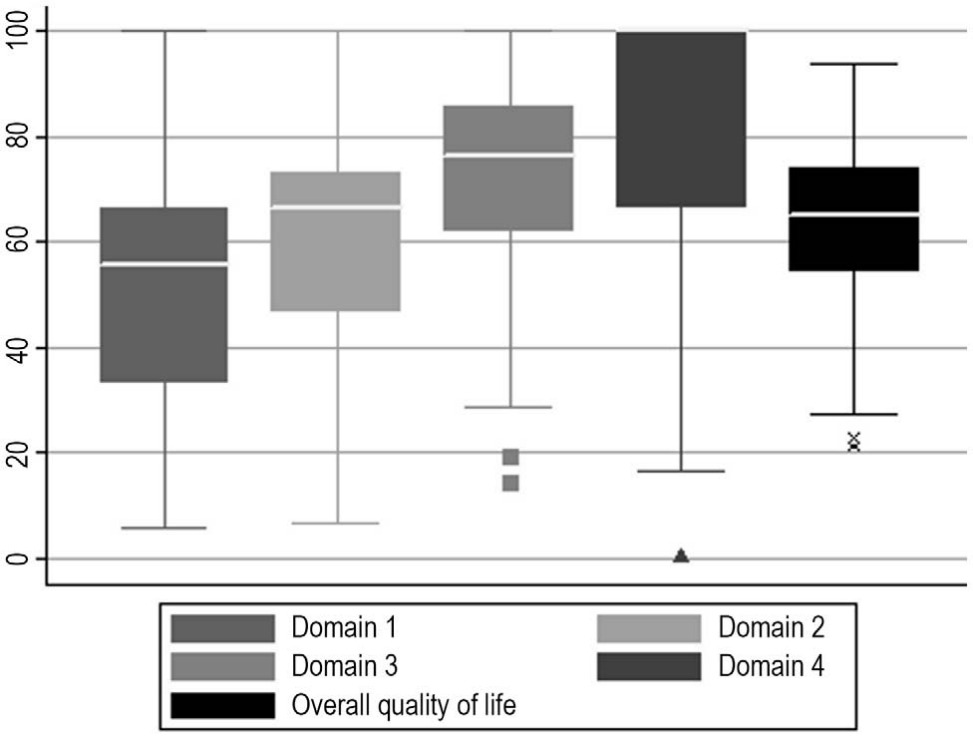
Box plots illustrating the different domain and overall quality of life scores as revealed in the present study. The study was carried out in June 2010, readily embedded in the second annual cross-sectional epidemiological survey of the Taabo health demographic surveillance system. The different domain and overall quality of life (QoL) scores were obtained through questionnaire-based QoL interviews with the study participants. The participants' scores were measured on a scale from 0 to 100, as detailed on the y-axis of the figure, with higher scores indicating higher wellbeing. Domain 1, environmental wellbeing; domain 2, psychological wellbeing; domain 3, physical wellbeing; domain 4, social wellbeing. Box plot: the ends of the box represent the 25^th^ and 75^th^ percentile of the scores; the middle line represents the median; the lower whisker represents the lowest value between the lower quartile and the lower quartile−1.5*(interquartile range); the upper whisker represents the highest value between the upper quartile and the upper quartile+1.5*(interquartile range); the small squares, triangles, and crosses indicate outliers.

Thirteen different field enumerators and supervisors of the Taabo HDSS were involved in interviewing the study participants, with no statistically significant inter-observer variation in reported mean QoL scores (*p* = 0.104). The mean summary score on QoL was 63.9 (range: 21.2–93.9) and Cronbach's alpha (0.805) indicated a good internal consistency of the QoL scores. The univariable linear regression model with the calculated QoL summary scores as outcome, and the QoL ratings directly expressed by the participants as explanatory variable, indicated a statistically significant positive correlation (*p*<0.001). The calculated scores on the four different domains and the summary score on QoL are illustrated in Figure 4. Generally lower scores were obtained for the domains comprising the participants' environment and psychological wellbeing, and higher scores for the physical and social wellbeing.

The mean domain and overall QoL scores in relation to sociodemographic and parasitological variables are shown in Table 4. The only statistically significant differences in the univariable analysis were an increased score in the environmental wellbeing of higher educated people (*p* = 0.043), those belonging to the wealthiest quintile (*p* = 0.006), and people with no hookworm infection (*p* = 0.002).

**Table 4:** Mean domain and overall quality of life scores in relation to sociodemographic determinants in rural Côte d'Ivoire.

Sociodemographic or	Mean domain and overall quality of life score									
parasitological determinants	Domain 1	*P*-value^a^	Domain 2	*P*-value^a^	Domain 3	*P*-value^a^	Domain 4	*P*-value^a^	Quality of life	*P*-value^a^
Sex: male	49.2		64.5		73.0		86.7		65.0	
Sex: female	52.1	0.275	60.5	0.180	69.5	0.267	83.7	0.496	62.8	0.385
Age: 18–40 years	48.9		63.5		74.0		87.6		65.2	
Age: 41–60 years	50.3		61.0		70.1		82.3		62.5	
Age: over 60 years	54.2	0.424	64.3	0.467	69.4	0.614	87.8	0.554	65.0	0.618
Education: no	51.1		62.7		68.9		87.8		63.3	
Education: primary school	45.9		62.6		76.6		83.8		64.7	
Education: secondary school	50.9		61.3		71.4		81.4		63.3	
Education: higher education	70.4	0.043*	68.9	0.859	77.0	0.423	80.6	0.234	73.0	0.393
Occupation: primary sector	48.3		61.9		70.5		84.8		62.8	
Occupation: secondary sector	56.9		64.2		70.8		91.7		66.1	
Occupation: tertiary sector	56.6	0.050	64.5	0.717	74.0	0.464	85.7	0.980	67.2	0.192
Socioeconomic status: most poor	47.2		64.1		75.3		91.0		65.4	
Socioeconomic status: very poor	46.6		59.8		72.6		82.4		62.6	
Socioeconomic status: poor	47.2		61.8		65.9		85.5		60.6	
Socioeconomic status: less poor	49.4		58.0		68.3		79.3		60.7	
Socioeconomic status: least poor	62.6	0.006*	69.0	0.091	74.4	0.071	87.8	0.392	70.3	0.063
*S. haematobium*: negative	50.6		62.8		71.7		85.8		64.2	
*S. haematobium*: positive	47.2	0.926	50.0	0.168	56.0	0.126	62.5	0.375	51.9	0.223
*S. mansoni:* negative	50.9		62.8		71.7		85.8		64.3	
*S. mansoni:* positive	33.3	0.251	50.0	0.191	56.0	0.114	62.5	0.180	47.7	0.130
Hookworm: negative	54.4		62.6		71.9		87.9		65.4	
Hookworm: positive	44.6	0.002*	62.6	0.895	70.5	0.702	81.3	0.143	61.6	0.114
*A. lumbricoides*: negative	50.6		62.6		71.3		85.3		63.9	
*A. lumbricoides*: positive	NA	NA	NA	NA	NA	NA	NA	NA	NA	NA
*T. trichiura*: negative	64.4		77.3		79.1		93.3		75.8	
*T. trichiura*: positive	50.2	0.070	62.2	0.105	71.1	0.233	85.1	0.434	63.6	0.060
*Plasmodium* spp.: negative	50.2		62.6		70.8		85.8		63.8	
*Plasmodium* spp.: positive	52.1	0.514	62.6	0.979	73.5	0.510	82.8	0.308	64.7	0.494

Mean domain and overall quality of life scores in relation to sociodemographic determinants among 187 adults in the Taabo health demographic surveillance system, south-central Côte d'Ivoire, in June 2010.

Domain 1, environmental wellbeing; domain 2, psychological wellbeing; domain 3, physical wellbeing; domain 4, social wellbeing.

NA, not applicable.

* = statistically significant (*p*<0.05).

a *P*-values from comparing mean scores by using Wilcoxon rank sum and Kruskal-Wallis tests as appropriate.

The wealth index (*p* = 0.034) and *S. mansoni* and *T. trichiura* infections of any intensity (*p* = 0.011 and *p* = 0.035, respectively) remained as the only three statistically significant explanatory variables for the overall QoL scores in the multivariable linear regression model with backward elimination (Table 5 and Table S1). The other three remaining variables after backward elimination were sex (*p* = 0.067), working in the secondary or tertiary sectors (*p* = 0.094), and hookworm infection of any intensity (*p* = 0.061). Whether age and wealth were used as continuous variables (e.g., age in years and wealth as wealth index as in the presented model) or as categorical variables (e.g., age as age categories and/or wealth as wealth quintiles; details on these models are not shown) influenced these findings only insofar as the individuals' occupation was also eliminated in the latter models.

**Table 5:** Associations remaining in the multivariable linear regression model after stepwise backward elimination.

Explanatory variable	Coeff.	95% CI	*P*-value
Sex^a^	−3.5	(−7.3, 0.2)	0.067
Working in secondary or tertiary sectors^b^	3.8	(−0.7, 8.3)	0.094
Wealth index^c^	1.2	(0.1, 2.3)	0.034*
*S. mansoni* infection of any intensity^d^	−16.4	(−29.2, −3.7)	0.011*
Hookworm infection of any intensity^e^	−3.9	(−8.0, 0.2)	0.061
*T. trichiura* infection of any intensity^f^	−12.6	(−24.4, −0.9)	0.035*

A multivariable linear regression model with a stepwise backward elimination procedure was adopted in order to identify those explanatory variables, which most significantly influence the study participants' quality of life (QoL) scores. The explanatory variables and indicators of the multivariable linear regression model at each step of the backward elimination procedure are shown in the supporting information (Table S1). The data on sociodemographic factors, parasitology, and QoL of the 187 study participants were collected in the Taabo health demographic surveillance system, south-central Côte d'Ivoire, in June 2010.

CI, confidence interval.

* = statistically significant (*p*<0.05).

a Reference category: male.

b Reference category: primary sector.

c Continuous variable.

d Reference category: no *S. mansoni* infection.

e Reference category: no hookworm infection.

f Reference category: no *T. trichiura* infection.

### Attrition Analysis

Comparison of participants who were included in the final analysis and participants who gave written informed consent and completed the questionnaire, but dropped out due to incomplete parasitological data, revealed no statistically significant differences in sociodemographic characteristics (Table 6) or mean domain and overall QoL scores (Table 7). Moreover, our attrition analysis revealed no significant difference between included and excluded people in terms of mean domain and overall QoL scores.

**Table 6:** Attrition analysis comparing sociodemographic determinants between included and excluded individuals.

Sociodemographic factor	Included	Excluded	*P*-value^a^
	(n = 187)	(n = 61)	
Sex: male	98	25	
Sex: female	89	36	0.121
Age: 18–40 years	66	27	
Age: 41–60 years	84	28	
Age: over 60 years	37	6	0.163
Education: no	100	34	
Education: primary school	39	18	
Education: secondary school	42	8	
Education: higher education	6	1	0.277
Occupation: primary sector	136	50	
Occupation: secondary sector	8	0	
Occupation: tertiary sector	43	11	0.164
Socioeconomic status: most poor	33	18	
Socioeconomic status: very poor	35	14	
Socioeconomic status: poor	42	8	
Socioeconomic status: less poor	39	10	
Socioeconomic status: least poor	38	11	0.189

The sociodemographic determinants of the 248 individuals who participated in the questionnaire survey were collected in the Taabo health demographic surveillance system, south-central Côte d'Ivoire, in June 2010.

a *P*-values from comparing the number of included individuals *vs.* the number of excluded individuals with a specific sociodemographic determinant by using χ^2^ and Fisher's exact test, as appropriate.

**Table 7:** Attrition analysis comparing mean domain and overall quality of life scores between included and excluded individuals.

Quality of life indicator	Included	Excluded	*P*-value^a^
	(n = 187)	(n = 61)	
Domain 1: environmental wellbeing	50.6	49.6	0.770
Domain 2: psychological wellbeing	62.6	60.9	0.464
Domain 3: physical wellbeing	71.3	68.0	0.213
Domain 4: social wellbeing	85.3	83.3	0.632
Overall quality of life	63.9	62.3	0.468

The domain and overall quality of life (QoL) scores of the 248 individuals who participated in the questionnaire survey were collected in the Taabo health demographic surveillance system, south-central Côte d'Ivoire, in June 2010.

a *P*-values from comparing the mean domain and overall QoL scores between included and excluded individuals by using Wilcoxon rank sum test.

## Discussion

We present an analysis from a QoL questionnaire survey conducted alongside the 2010 cross-sectional epidemiological survey and deworming campaign in the Taabo HDSS in south-central Côte d'Ivoire. Results of a multivariable linear logistic regression model revealed that adults' QoL is reduced considerably among those infected with different species of helminths, regardless of the intensity of infection. Indeed, we found that the perceived QoL among adults infected with *S. mansoni* and *T. trichiura* was 16 points (95% confidence interval (CI): 4–29 points) and 13 points (95% CI: 1–24 points) lower on a scale from 0 to 100 than the reported QoL of non-infected individuals. The only other statistically significant effect was a 1-point (95% CI: 0.1–2 points) increase in QoL per one unit increase in the wealth index. Other important explanatory variables that remained in our multivariable linear regression model after applying a stepwise backward elimination procedure were sex, indicating a 4 points (95% CI: −7–0.2 points) decrease in QoL of females; occupation, indicating a 4 points (95% CI: −0.7–8 points) increase in QoL of those working mainly in the secondary or tertiary sectors; and hookworm infections, indicating a 4 points (95% CI: −8–0.2 points) decrease in QoL of those infected.

Our results have to be interpreted with caution, but raise many interesting issues. As a first critical point, it has to be considered that the sampling of the current study depended on a stratified random sampling. Starting in mid-2009, our team pursued a yearly cross-sectional epidemiological survey among approximately 7% of the people who were under demographic and health surveillance in the Taabo HDSS. The present study was linked to the June 2010 cross-sectional survey, using a sub-sample (i.e., all head of households plus a second randomly selected person of the same household but the opposite sex to maintain gender balance). Given our sampling approach and in view of operational and financial considerations, no formal sample size calculation was made for the present study.

Second, our final sample size of 187 individuals was relatively small and the compliance rate of 73.3% suboptimal. However, somewhat higher drop-out rates had to be expected as the participants were adults, many of whom were illiterate. Compared to school-aged children, adults seemed to be somewhat reluctant or ashamed to provide any stool or urine samples. Importantly though, the attrition analysis revealed no statistically significant differences in the available indicators between the included and excluded adults, and hence no selection bias seems to have been introduced by the drop-outs.

Third, the absence of a statistically significant inter-observer variation suggests that our questionnaire results are reliable. Cronbach's alpha as well as the highly significant positive correlation between the calculated summary scores on QoL based on all questions and the QoL ratings directly expressed by the participants in the final question of the questionnaire indicate internal consistency and validity of the QoL scores.

Fourth, the parasitological diagnosis was based on single stool and urine samples with duplicate Kato-Katz thick smear examinations and single urine filtration, respectively. There is a large body of work demonstrating that multiple sampling or a combination of diagnostic methods result in more accurate diagnosis [29], [30]. It follows that we missed some helminth infections, particularly those of light intensity. At this stage, it is difficult to say how these false negative results might have influenced our findings. However, assuming that helminth infections have no beneficial impact on patients' QoL, one hypothesis would be that the false negative results mistakenly lowered the QoL of the uninfected comparison group in our study. Furthermore, assuming that most individuals with false negative results suffered from light infections (like most of those effectively diagnosed as infected), a next hypothesis is that false negative results do not systematically distort the QoL of the infected comparison group. This hypothesis would even hold true when expecting a correlation between infection intensity and QoL. One could therefore argue that the false negative results may have mistakenly reduced the measured QoL difference between the uninfected and infected comparison group in our study.

Fifth, the mean domain and overall QoL scores displayed in Table 4 were consistently lower in participants with helminth infections compared to their helminth-free counterparts. For instance, participants tested positive for *S. mansoni* reported a statistically non-significant, but consistent decrease in the mean environmental (*p* = 0.251), psychological, physical, and social wellbeing (all *p*<0.2). This remarkable consistency increases the plausibility of the presented findings. However, with regard to *Plasmodium* spp. infections the picture was less clear. Participants with positive malaria RDTs reported higher mean scores on environmental and physical wellbeing and had higher overall QoL score. These counterintuitive findings might at least partially be explained by false positive results of the RDTs because of delayed clearance of the circulating antigen [31] and by the acquired semi-immunity of probably all examined adults leading to usually uncomplicated malaria with mild symptoms [32].

Sixth, the effects of the sociodemographic determinants on the mean domain and overall QoL scores were somewhat less clear in the univariable comparison as summarized in Table 4. However, the statistically significantly higher scores in environmental wellbeing of higher educated adults and the wealthiest participants demonstrate also the unsurprising importance of sociodemographic determinants. This statement is supported by the fact that sex, the sector of occupation, and the wealth index also remained in the multivariable linear regression model after the stepwise backward elimination.

Seventh, there were only a few people infected with *S. haematobium*, *S. mansoni*, and *T. trichiura* in our study sample and even though the probably most obvious confounders (e.g., age, sex, educational attainment, occupation, and socioeconomic status) were included in our analyses, we cannot rule out an effect of other potential confounders (e.g., lack of access to clean water and sanitation). Furthermore, we did not consider infection intensities or explore interaction terms for combined infections in our multivariable linear regression model as more complex modeling was not possible due to the comparatively small numbers.

Eighth, the here presented decrease of 16 points on the 0 to 100 QoL scale due to *S. mansoni* infections can be interpreted as a disability of 16%. This disability estimate is slightly below previously presented results from QoL surveys on advanced, chronic *S. japonicum* in the People's Republic of China, which revealed a mean disability of 19% [7] and up to even 45% [10]. However, our results are considerably higher than current WHO estimates, which indicate a disability of only 1% for any *Schistosoma* infection and 10% for advanced renal or hepatic infection [33]. Unfortunately, no QoL surveys for comparison could be identified with regard to soil-transmitted helminth infections. However, if the 13 points reduction for *T. trichiura* infections and the 4 points reduction for hookworm infections are also considered as disabilities of 13% and 4%, they are in the range of the disability weights listed by WHO (trichuriasis-associated high intensity infection 0%, contemporaneous cognitive deficit 1%, massive dysentery syndrome 12%, and cognitive impairment 2%; hookworm-associated high intensity infection 1%, anemia 2%, and cognitive impairment 2%) [33].

In conclusion, we found consistent and significant results on the effect of schistosomiasis, soil-transmitted helminthiasis, and sociodemographic determinants on adults' QoL in rural Côte d'Ivoire. It is conceivable that helminth-infected adults in the present study suffered from advanced chronic infections and therefore reported notable losses in QoL. Our results warrant further investigation on the disability induced by helmintic infections and further probing of the usefulness and applicability of generic QoL questionnaires in this regard. Future studies should adhere to a more rigorous sampling strategy and sample size calculation, optimally in a randomized trial design, which allows for an improved control of potential confounders and the assessment of interactions due to combined infections. Furthermore, they should consider additional qualitative research to further explore the local residents' concept about QoL, additional verification of the QoL questionnaire's reliability and validity (e.g., test-retest comparison, comparison of questionnaire results with objectively measurable indicators [9]), more intensive parasitological diagnosis (e.g., repeated stool, urine, and blood sampling, multiple testing, and concurrent use of different diagnostic methods), further analyses regarding the effects of differing infection intensities, and clinical examinations (e.g., stethoscopy and ultrasound) to confirm chronic sequelae and other medical complaints.

## Supporting Information

Alternative Language Abstract S1 **Translation of the Abstract into French by Kigbafori D. Silué and Eliézer K. N'Goran.**
(DOC)

Text S1 **Questionnaire for evaluating the health state of individuals (in French).**
(DOC)

Text S2 **Questionnaire for evaluating the health state of individuals (unofficial translation into English).**
(DOC)

Table S1 **Explanatory variables and indicators of the multivariable linear regression model at each step of the backward elimination procedure.**
(DOC)
