# Supplementary material for: Schistosomiasis, Soil-Transmitted Helminthiasis, and Sociodemographic Factors Influence Quality of Life of Adults in Côte d'Ivoire
Source: PLoS Negl Trop Dis. 2012 Oct 4;6(10):e1855. doi: 10.1371/journal.pntd.0001855 (PMC3464303; doi:10.1371/journal.pntd.0001855)
Supplement: Alternative Language Abstract S1 — Translation of the Abstract into French by Kigbafori D. Silué and Eliézer K. N'Goran. (DOC) [file pntd.0001855.s001.doc]

**Schistosomiase, géohelminthiases et facteurs sociodémographiques influencent la qualité de vie des adultes en Côte d’Ivoire**

**Résumé**

***Contexte:*** Les estimations du fardeau attribué aux différentes maladies sont aujourd’hui largement utilisées en santé publique pour établir les priorités. Parmi ces indicateurs, le nombre d’années de vie corrigées du facteur d’invalidité (AVCI) est le plus couramment employé. Cependant, cet indicateur fait l’objet d’une critique continue du fait que pour le calculer, l’estimation de l’invalidité subie par un patient est essentielle et cette dernière représente un sujet encore largement débattu.

***Méthodologie:*** Une enquête épidémiologique transversale a été conduite dans le site de surveillance démographique et de santé (SSDS) établi en 2008 à Taabo, au centre sud de la Côte d’Ivoire. Cette enquête avait pour objet à partir de données collectées au niveau de la population, de rechercher de nouvelles preuves sur le handicap causé par la schistosomiase et les géohelminthiases. Les résultats des examens parasitologiques de selles, d’urine et de sang ont été associés aux résultats d’un questionnaire sur la qualité de vie de 187 adultes. Un modèle de régression linéaire multiple a été utilisé pour identifier les associations significatives, en considérant aussi des caractéristiques sociodémographiques obtenues à partir de la base de données du SSDS de Taabo.

***Principaux Résultats:*** Les prévalences des ankylostomes, *Plasmodium* spp, *Trichuris trichiura*, *Schistosoma haematobium* et *Schistosoma mansoni* ont été de 39,0 %, 18,2 %, 2,7 %, 2,1 % et 2,1 %, respectivement. Les infestations par *S. mansoni* et *T. trichiura* de toute intensité, réduisent l’auto-évaluation de la qualité de vie des participants de 16 points (intervalle de confidence (IC) à 95 %: 4-29 points) et 13 points (IC 95 %: 1-24 points) respectivement sur une échelle de 0 (la plus mauvaise qualité de vie) à 100 points (la mieux qualité de vie). Le seul autre effet statistiquement significatif était une augmentation de 1 point (IC 95 %: 0.1-2 points) sur l’échelle, qui mesure la qualité de vie, si un indice de richesse calculé par nous-même augmentait d’une unité.

***Conclusion:*** En considérant aussi des caractéristiques sociodémographiques, nous avons montré sur la base d’associations statistiquement et significatives, l’impact négatif de la schistosomiase et des géohelminthiases sur l’auto-évaluation de la qualité de vie chez les adultes. Ces résultats démontrent la pertinence d’un questionnaire générique sur la qualité de la vie dans un tel contexte et incitent à une enquête plus approfondie sur le handicap induit par helminthiases.

***Traduction:*** Kigbafori D. Silué et Eliézer K. N’Goran
