# Supplementary material for: Schistosomiasis, Soil-Transmitted Helminthiasis, and Sociodemographic Factors Influence Quality of Life of Adults in Côte d'Ivoire
Source: PLoS Negl Trop Dis. 2012 Oct 4;6(10):e1855. doi: 10.1371/journal.pntd.0001855 (PMC3464303; doi:10.1371/journal.pntd.0001855)
Supplement: Text S1 — Questionnaire for evaluating the health state of individuals (in French). (DOC) [file pntd.0001855.s002.doc]

## Enquête pour évaluer l’état de santé

## Questionnaire pour les participents à l’enquête transversal au SSD de Taabo en Juin 2010.

Cher(e) participant(e),

Nous avons rédigé quelques questions concernant votre avis sur votre état de santé et vos activités quotidiennes. Ces questions ne sont pas un test avec des bonnes et des mauvaises réponses. Pour nous, il est plus important de savoir comment vous allez et seule votre opinion personnelle et honnête nous intéresse. Nous vous prions donc de nous aider à remplir le questionnaire complètement.

| **Village/Campement :** | | | | | | | | |
| --- | --- | --- | --- | --- | --- | --- | --- | --- |
| **Date :** | **Nom de l’enquêteur :** | | | | | | | |
| **Numéro d’identification du participant** | |  |  |  |  |  |  |  |

# Facteur de risque (6)

Indiquez le numéro correct (1, 2 ou 9) pour chaque réponse prévue à la gauche.

| faire la lessive1) Quelles activités liées à l’eau (de rivières, lacs, marigot etc.) avez-vous pratiqué durant ces 4 dernières semaines ?  |  | | --- | | faire la vaisselle | | se laver | | nager | | cuisiner | | laver les enfants | | traverser les rivières | | pêcher au filet | | pêcher à l’hameçon | | cultiver du riz | | pratiques religieuses | | autres :……………………….. | |  |  (1=oui / 2=non / 9=ne sait pas) | |  | | --- | |  | |  | |  | |  | |  | |  | |  | |  | |  | |  | |  | |
| --- | --- | --- | --- | --- | --- | --- | --- | --- | --- | --- | --- | --- | --- | --- | --- | --- | --- | --- | --- | --- | --- | --- | --- | --- | --- | --- |
| **2) Quelle eau buvez-vous ?**  (1=oui / 2=non / 9=ne sait pas)   | eau de marigot, ruisseau, rivière | | --- | | eau de pluie | | eau courrant / robinet | | eau en bouteille / eau minérale / awa | | autres :……………………….. | | |  | | --- | |  | |  | |  | |  | |
| **3) Ces 4 dernières semaines, avez-vous utilisé…**  (1=oui / 2=non / 9=ne sait pas)   | une moustiquaire | | --- | | des insecticides (p. ex. timor) | | moustico | | autres :……………………….. | | |  | | --- | |  | |  | |  | |
| **4) Est-ce que vous utilisez du savon pour…**   | faire la lessive | | --- | | faire la vaisselle | | vous laver | | vous laver les mains avant de manger | | vous laver les mains après les selles | | laver les mains au retour du travail |   (1=oui / 2=non / 9=ne sait pas) | |  | | --- | |  | |  | |  | |  | |  | |
| **5) De temps en temps, mangez-vous…**  (1=oui / 2=non / 9=ne sait pas)   | de la viande crue | | --- | | des poisons crus | | des fruits crus | | des légumes crus | | |  | | --- | |  | |  | |  | |
| **6) Quels animaux possédez-vous ?**  (1=oui / 2=non / 9=ne sait pas)   | chien | | --- | | poule / volaille | | lapin | | cobaye | | cochon | | cabri | | mouton | | vache / buffle | | autres :………………………… | | |  | | --- | |  | |  | |  | |  | |  | |  | |  | |  | |

# Signes & Symptômes (2)

| **7) Avez-vous expérimenté un ou plusieurs signes ou symptômes suivants durant ces 4 dernières semaines?**   | sang dans les selles | | --- | | sang dans l’urine | | mal de ventre | | ballonnement du ventre | | perte de l’appétit | | mal de tête | | troubles visuels | | problème pour articuler/ parler | | fièvre/ corps qui chauffe | | faiblesse/ fatigue | | vomissement | | vertige | | diarrhée | | douleur en pissant | | perte de poids | | boutons ou tache sur la peau | | mal à la poitrine | | problème pour respirer | | toux | | corps qui gratte | | partie du corps enflée | | mal de rein | | autres :…………………. |   (1=oui / 2=non / 9=ne sait pas) | |  | | --- | |  | |  | |  | |  | |  | |  | |  | |  | |  | |  | |  | |  | |  | |  | |  | |  | |  | |  | |  | |  | |  | |  | |
| --- | --- | --- | --- | --- | --- | --- | --- | --- | --- | --- | --- | --- | --- | --- | --- | --- | --- | --- | --- | --- | --- | --- | --- | --- | --- | --- | --- | --- | --- | --- | --- | --- | --- | --- | --- | --- | --- | --- | --- | --- | --- | --- | --- | --- | --- | --- | --- |
| **8) Quelles sont les maladies fréquentes dans votre ménage ?**   | Bilharziose ou schistosomiase | | --- | | Tuberculose | | Diarrhée | | Onchocercose | | VIH / SIDA | | Méningite | | Filariose / éléphantiasis | | Paludisme | | Ulcère de buruli | | Vers intestinaux | | Lèpre | | Je ne connais pas ces maladies |   (1=oui / 2=non / 9= ne sait pas) | |  | | --- | |  | |  | |  | |  | |  | |  | |  | |  | |  | |  | |  | |

# Qualité de vie (adultes : WHOQOL bref (24))

Marquez la réponse qui est la plus vraie pour chaque question.

| **9) Vous sentez-vous en bonne santé ?** | Non, pas du tout | Plus ou moins | Oui, la plupart du temps | Oui, absolument |
| --- | --- | --- | --- | --- |
| **10) Avez vous eu assez d’énergie durant ces 4 dernières semaines pour mener votre vie quotidienne ?** | Non, pas du tout | Plus ou moins | Oui, la plupart du temps | Oui, absolument |
| **11) Avez-vous eu assez d’argent pour acheter tout ce dont vous aviez besoin durant ces 4 dernières semaines ?** | Non, pas du tout | Plus ou moins | Oui, la plupart du temps | Oui, absolument |
| **12) Avez-vous eu assez de temps libre durant ces 4 dernières semaines ?** | Non, pas du tout | Plus ou moins | Oui, la plupart du temps | Oui, absolument |
| **13) Êtes-vous satisfait de votre capacité de travail ?** | Non, pas du tout | Plus ou moins | Oui, la plupart du temps | Oui, absolument |
| **14) Êtes-vous satisfait de vos relations personnelles (par exemple avec vos amis ou votre famille) ?** | Non, pas de tout | Plus ou moins | Oui, la plupart du temps | Oui, absolument |
| **15) Êtes-vous satisfait du support de vos amis ?** | Non, pas de tout | Plus ou moins | Oui, la plupart du temps | Oui, absolument |
| **16) Êtes-vous satisfait de votre logement ?** | Non, pas de tout | Plus ou moins | Oui, la plupart du temps | Oui, absolument |
| **17) Êtes-vous satisfait de votre accès au centre de santé le plus proche ?** | Non, pas de tout | Plus ou moins | Oui, la plupart du temps | Oui, absolument |
| **18) Êtes-vous satisfait de vos possibilités de transport ?** | Non, pas de tout | Plus ou moins | Oui, la plupart du temps | Oui, absolument |
| **19) Êtes-vous content avec vous et votre situation en générale ?** | Non, pas de tout | Plus ou moins | Oui, la plupart du temps | Oui, absolument |

| **20) Combien de fois avez-vous pris des médicaments durant ces 4 dernières semaines ?** | | Jamais | | Rarement | | Souvent | | Toujours |
| --- | --- | --- | --- | --- | --- | --- | --- | --- |
| **21) Combien de fois vous êtes-vous réjouit de votre vie durant ces 4 dernières semaines?** | | Jamais | | Rarement | | Souvent | | Toujours |
| **22) Combien de fois avez-vous pensé que votre vie avait un sens durant ces 4 dernières semaines?** | | Jamais | | Rarement | | Souvent | | Toujours |
| **23) Combien de fois avez-vous eu des problèmes pour vous concentrer durant ces 4 dernières semaines ?** | Jamais | | Rarement | | Souvent | | Toujours | |
| **24) Combien de fois ne vous êtes vous pas sentis en sécurité durant ces 4 dernières semaines?** | | Jamais | | Rarement | | Souvent | | Toujours |
| **25) Combien de fois avez-vous eu des sentiments négatifs, comme par exemple la peur, le désespoir, la tristesse ou la dépression ?** | | Jamais | | Rarement | | Souvent | | Toujours |

| **26) Avez vous souffert de douleurs, durant ces 4 dernières semaines, qui vous ont handicapées dans vos activités quotidiennes ?** | Non,  jamais | Oui, mais rarement | Oui, souvent | Oui, toujours |
| --- | --- | --- | --- | --- |
| **27) Avez-vous eu de la peine à marcher durant ces 4 dernières semaines?** | Non,  jamais | Oui, mais rarement | Oui, souvent | Oui, toujours |
| **28) Avez-vous bien dormi durant ces 4 dernières semaines?** | Non,  jamais | Oui, mais rarement | Oui, souvent | Oui, toujours |
| **29) Avez-vous eu des problèmes pour exécuter vos activités quotidiennes durant ces 4 dernières semaines?** | Non,  jamais | Oui, mais rarement | Oui, souvent | Oui, toujours |

| **30) Comment jugez-vous votre qualité de vie, en général ?** | Très bien | Bien | Mal | Très mal |
| --- | --- | --- | --- | --- |

Merci beaucoup de votre merveilleuse collaboration!!!

☺
