# Supplementary material for: Schistosomiasis, Soil-Transmitted Helminthiasis, and Sociodemographic Factors Influence Quality of Life of Adults in Côte d'Ivoire
Source: PLoS Negl Trop Dis. 2012 Oct 4;6(10):e1855. doi: 10.1371/journal.pntd.0001855 (PMC3464303; doi:10.1371/journal.pntd.0001855)
Supplement: Text S2 — Questionnaire for evaluating the health state of individuals (unofficial translation into English). (DOC) [file pntd.0001855.s003.doc]

## Survey to evaluate the health status

## Questionnaire for participants of the cross-sectional survey in the Taabo HDSS in June 2010

Dear participant,

We prepared some questions regarding your opinion about your personal health status and daily activities. These questions are not a test with right or wrong answers. For us, it is most important to know how you feel and we are only interested in your personal and honest opinion. We would therefore like to ask you to help us complete the following questionnaire.

| **Name of village/hamlet:** | | | | | | | | |
| --- | --- | --- | --- | --- | --- | --- | --- | --- |
| **Date:** | **Name of field enumerator:** | | | | | | | |
| **ID number of participant** | |  |  |  |  |  |  |  |

# Risk Factors (6)

Note the correct number (1, 2, or 9) for each response indicated on the left.

| do the laundry1) Which water-related (rivers, lakes, ponds, etc.) activities did you do during the last 4 weeks?  |  | | --- | | wash the dishes | | wash yourself | | swimming | | cooking | | wash the children | | cross the rivers | | fishing with net | | fishing with fishing-rod | | cultivate rice | | religious worship | | others:……………………….. | |  |  (1=yes / 2=no / 9=don’t know) | |  | | --- | |  | |  | |  | |  | |  | |  | |  | |  | |  | |  | |  | |
| --- | --- | --- | --- | --- | --- | --- | --- | --- | --- | --- | --- | --- | --- | --- | --- | --- | --- | --- | --- | --- | --- | --- | --- | --- | --- | --- |
| **2) Which water do you drink?**  (1=yes / 2=no / 9=don’t know)   | water from rivers, lakes, ponds | | --- | | rain water | | tap water | | bottled water / mineral water / Awa | | others:……………………….. | | |  | | --- | |  | |  | |  | |  | |
| **3) During the last 4 weeks, did you use…**  (1=yes / 2=no / 9=don’t know)   | a mosquito net | | --- | | insecticides (e.g. Timor) | | moustico | | others:……………………….. | | |  | | --- | |  | |  | |  | |
| **4) Do you use soap for…**   | doing the laundry | | --- | | washing the dishes | | washing yourself | | washing your hands before eating | | washing your hands after defecation | | washing your hands when returning from work |   (1=yes / 2=no / 9=don’t know) | |  | | --- | |  | |  | |  | |  | |  | |
| **5) From time to time, do you eat…**  (1=yes / 2=no / 9=don’t know)   | uncooked meat | | --- | | uncooked fish | | uncooked fruits | | uncooked vegetables | | |  | | --- | |  | |  | |  | |
| **6) What animals do you own?**  (1=yes / 2=no / 9=don’t know)   | dog | | --- | | chicken / poultry | | rabbit | | guinea pig | | pig | | goat | | sheep | | cow / buffalo | | others:………………………… | | |  | | --- | |  | |  | |  | |  | |  | |  | |  | |  | |

# Signs & Symptoms (2)

| **7) Did you suffer from one or several of the following signs and symptoms during the last 4 weeks?**   | blood in the stool | | --- | | blood in the urine | | abdominal pain | | flatulence | | loss of appetite | | headache | | impaired vision | | problems to articulate / speak | | fever / hot body | | weakness / fatigue | | vomiting | | vertigo | | diarrhea | | pain when urinating | | weight loss | | spots or pimples on the skin | | chest pain | | problems to breathe | | cough | | itching body | | part of the body swollen | | kidney pain | | others :…………………. |   (1=yes / 2=no / 9=don’t know) | |  | | --- | |  | |  | |  | |  | |  | |  | |  | |  | |  | |  | |  | |  | |  | |  | |  | |  | |  | |  | |  | |  | |  | |  | |
| --- | --- | --- | --- | --- | --- | --- | --- | --- | --- | --- | --- | --- | --- | --- | --- | --- | --- | --- | --- | --- | --- | --- | --- | --- | --- | --- | --- | --- | --- | --- | --- | --- | --- | --- | --- | --- | --- | --- | --- | --- | --- | --- | --- | --- | --- | --- | --- |
| **8) What are frequent diseases in your household?**   | Bilharzia or schistosomiasis | | --- | | Tuberculosis | | Diarrhea | | Onchocerciasis | | HIV / AIDS | | Meningitis | | Filariasis/elephantiasis | | Malaria | | Buruli ulcer | | Intestinal helminths | | Leprosy | | I don’t know these diseases |   (1=yes / 2=no / 9=don’t know) | |  | | --- | |  | |  | |  | |  | |  | |  | |  | |  | |  | |  | |  | |

# Quality of life (adults: WHOQOL bref (24))

Mark the most appropriate response for each question.

| **9) Do you feel that you are in good health?** | No, not  at all | More or less | Yes, most of the time | Yes, absolutely |
| --- | --- | --- | --- | --- |
| **10) Did you have enough energy to complete your daily tasks during the last 4 weeks?** | No, not  at all | More or less | Yes, most of the time | Yes, absolutely |
| **11) Did you have enough money to buy all things that you needed during the last 4 weeks?** | No, not  at all | More or less | Yes, most of the time | Yes, absolutely |
| **12) Did you have enough leisure time during the last 4 weeks?** | No, not  at all | More or less | Yes, most of the time | Yes, absolutely |
| **13) Are you satisfied with your working capacity?** | No, not  at all | More or less | Yes, most of the time | Yes, absolutely |
| **14) Are you satisfied with your personal relationships (for instance with your friends or family)?** | No, not  at all | More or less | Yes, most of the time | Yes, absolutely |
| **15) Are you satisfied with the support of your friends?** | No, not  at all | More or less | Yes, most of the time | Yes, absolutely |
| **16) Are you satisfied with your housing?** | No, not  at all | More or less | Yes, most of the time | Yes, absolutely |
| **17) Are you satisfied with the accessibility of the nearest health center?** | No, not  at all | More or less | Yes, most of the time | Yes, absolutely |
| **18) Are you satisfied with the means of transportation that are available for you?** | No, not  at all | More or less | Yes, most of the time | Yes, absolutely |
| **19) In general, are you satisfied with you and your situation?** | No, not  at all | More or less | Yes, most of the time | Yes, absolutely |

| **20) How often did you take drugs during the last 4 weeks?** | | Never | | Rarely | | Often | | Always |
| --- | --- | --- | --- | --- | --- | --- | --- | --- |
| **21) How often were you happy about / did you enjoy your life during the last 4 weeks?** | | Never | | Rarely | | Often | | Always |
| **22) How often did you think that your life makes sense / is meaningful during the last 4 weeks?** | | Never | | Rarely | | Often | | Always |
| **23) How often did you have problems to concentrate yourself during the last 4 weeks?** | Never | | Rarely | | Often | | Always | |
| **24) How often did you feel unsafe during the last 4 weeks?** | | Never | | Rarely | | Often | | Always |
| **25) How often did you have negative feelings, like anxiety, despair, sadness or depression during the last 4 weeks?** | | Never | | Rarely | | Often | | Always |

| **26) Did you suffer from physical pain, which prevented you from doing what you needed or wanted to do during the last 4 weeks?** | No,  never | Yes, but rarely | Yes,  often | Yes,  always |
| --- | --- | --- | --- | --- |
| **27) Did you suffer from physical pain when walking around during the last 4 weeks?** | No,  never | Yes, but rarely | Yes,  often | Yes,  always |
| **28) Did you sleep well during the last 4 weeks?** | No,  never | Yes, but rarely | Yes,  often | Yes,  always |
| **29) Did you have any problems to perform your daily living activities during the last 4 weeks?** | No,  never | Yes, but rarely | Yes,  often | Yes,  always |

| **30) How would you rate your quality of life in general?** | Very good | Good | Bad | Very bad |
| --- | --- | --- | --- | --- |

Thank you very much for your wonderful collaboration!!!

☺
