# Supplementary material for: Schistosomiasis, Soil-Transmitted Helminthiasis, and Sociodemographic Factors Influence Quality of Life of Adults in Côte d'Ivoire
Source: PLoS Negl Trop Dis. 2012 Oct 4;6(10):e1855. doi: 10.1371/journal.pntd.0001855 (PMC3464303; doi:10.1371/journal.pntd.0001855)
Supplement: Table S1 — Explanatory variables and indicators of the multivariable linear regression model at each step of the backward elimination procedure. (DOC) [file pntd.0001855.s004.doc]

**Table S1: Explanatory variables and indicators of the multivariable linear regression model at each step of the backward elimination procedure.**

| **Explanatory variables and indicators of the** | **Full model** | |  | **Step 1: eliminate** | |  | **Step 2: combine** | |  | **Step 3: combine** | |  | **Step 4: eliminate** | |
| --- | --- | --- | --- | --- | --- | --- | --- | --- | --- | --- | --- | --- | --- | --- |
| **multivariable linear regression model** |  | |  | **age** | |  | **education** | |  | **education** | |  | **education** | |
|  | **Coeff.** | ***p-value*** |  | **Coeff.** | ***p-value*** |  | **Coeff.** | ***p-value*** |  | **Coeff.** | ***p-value*** |  | **Coeff.** | ***p-value*** |
| Sex^a^ | -3.738 | 0.063 |  | -3.717 | 0.064 |  | -3.636 | 0.071 |  | -3.388 | 0.089 |  | -3.467 | 0.072 |
| Age in years^b^ | -0.025 | 0.721 |  | --- | --- |  | --- | --- |  | --- | --- |  | --- | --- |
| Education: primary school^c^ | 1.169 | 0.638 |  | 1.298 | 0.596 |  | 1.233 | 0.618 |  | 0.355 | 0.871 |  | --- | --- |
| Education: secondary school^c^ | -1.639 | 0.569 |  | -1.516 | 0.598 |  | -1.119 | 0.694 |  | --- | --- |  | --- | --- |
| Education: higher education^c^ | 3.558 | 0.552 |  | 3.901 | 0.510 |  | --- | --- |  | --- | --- |  | --- | --- |
| Occupation: secondary sector^d^ | 5.990 | 0.198 |  | 5.884 | 0.205 |  | 5.860 | 0.208 |  | 6.255 | 0.177 |  | 6.272 | 0.176 |
| Occupation: tertiary sector^d^ | 3.513 | 0.177 |  | 3.400 | 0.187 |  | 3.597 | 0.163 |  | 3.086 | 0.218 |  | 3.176 | 0.193 |
| Wealth index^b^ | 1.201 | 0.044 |  | 1.198 | 0.045 |  | 1.288 | 0.029 |  | 1.227 | 0.036 |  | 1.242 | 0.030 |
| *S. haematobium* infection of any intensity^e^ | -3.903 | 0.557 |  | -4.021 | 0.544 |  | -3.827 | 0.564 |  | -3.777 | 0.570 |  | -3.760 | 0.572 |
| *S. mansoni* infection of any intensity^f^ | -16.314 | 0.017 |  | -16.010 | 0.018 |  | -16.725 | 0.014 |  | -16.335 | 0.016 |  | -16.428 | 0.015 |
| Hookworm infection of any intensity^g^ | -3.621 | 0.085 |  | -3.651 | 0.082 |  | -3.853 | 0.066 |  | -3.933 | 0.061 |  | -3.941 | 0.060 |
| *T. trichiura* infection of any intensity^h^ | -12.125 | 0.043 |  | -12.089 | 0.043 |  | -12.347 | 0.040 |  | -12.441 | 0.038 |  | -12.399 | 0.039 |
| *Plasmodium* spp. infection of any intensity^i^ | 1.539 | 0.533 |  | 1.506 | 0.541 |  | 1.531 | 0.536 |  | 1.489 | 0.547 |  | 1.485 | 0.548 |
| Constant | 80.087 | <0.001 |  | 78.895 | <0.001 |  | 79.259 | <0.001 |  | 79.210 | <0.001 |  | 79.349 | <0.001 |
| Akaike information criterion of the model | 1524.883 | --- |  | 1523.007 | --- |  | 1521.939 | --- |  | 1520.571 | --- |  | 1518.596 | --- |
| Likelihood ratio test p-value at this step | --- | --- |  | --- | 0.724 |  | --- | 0.334 |  | --- | 0.427 |  | --- | 0.875 |

A multivariable linear regression model with a stepwise backward elimination procedure was adopted in order to identify those explanatory variables, which most significantly influence the study participants’ quality of life scores. The data on sociodemographic factors, parasitology, and quality of life of the 187 study participants were collected in the Taabo health demographic surveillance system, south-central Côte d’Ivoire, in June 2010.

^a^ Reference category: male.

^b^ Continuous variable.

^c^ Reference category: no education.

^d^ Reference category: primary sector.

^e^ Reference category: no *S. haematobium* infection.

^f^ Reference category: no *S. mansoni* infection.

^g^ Reference category: no hookworm infection.

^h^ Reference category: no *T. trichiura* infection.

^i^ Reference category: no *Plasmodium* spp. infection.

**Table S1: Continued.**

| **Explanatory variables and indicators of the** | **Step 5: eliminate** | |  | **Step 6: eliminate** | |  | **Step 7: combine** | |  | **Final model** | |
| --- | --- | --- | --- | --- | --- | --- | --- | --- | --- | --- | --- |
| **multivariable linear regression model** | ***S. haematobium*** | |  | ***Plasmodium* spp.** | |  | **occupation** | |  | **(see also Table 5)** | |
|  | **Coeff.** | ***p-value*** |  | **Coeff.** | ***p-value*** |  | **Coeff.** | ***p-value*** |  | **Coeff.** | ***p-value*** |
| Sex^a^ | -3.551 | 0.065 |  | -3.575 | 0.064 |  | -3.528 | 0.067 |  | -3.528 | 0.067 |
| Age in years^b^ | --- | --- |  | --- | --- |  | --- | --- |  | --- | --- |
| Education: primary school^c^ | --- | --- |  | --- | --- |  | --- | --- |  | --- | --- |
| Education: secondary school^c^ | --- | --- |  | --- | --- |  | --- | --- |  | --- | --- |
| Education: higher education^c^ | --- | --- |  | --- | --- |  | --- | --- |  | --- | --- |
| Occupation: secondary sector^d^ | 6.003 | 0.193 |  | 6.189 | 0.179 |  | 3.811 | 0.094 |  | 3.811 | 0.094 |
| Occupation: tertiary sector^d^ | 3.265 | 0.179 |  | 3.300 | 0.175 |  | --- | --- |  | --- | --- |
| Wealth index^b^ | 1.267 | 0.027 |  | 1.249 | 0.029 |  | 1.207 | 0.034 |  | 1.207 | 0.034 |
| *S. haematobium* infection of any intensity^e^ | --- | --- |  | --- | --- |  | --- | --- |  | --- | --- |
| *S. mansoni* infection of any intensity^f^ | -17.153 | 0.009 |  | -17.077 | 0.009 |  | -16.420 | 0.011 |  | -16.420 | 0.011 |
| Hookworm infection of any intensity^g^ | -3.972 | 0.058 |  | -3.925 | 0.061 |  | -3.932 | 0.061 |  | -3.932 | 0.061 |
| *T. trichiura* infection of any intensity^h^ | -12.423 | 0.038 |  | -12.682 | 0.034 |  | -12.623 | 0.035 |  | -12.623 | 0.035 |
| *Plasmodium* spp. infection of any intensity^i^ | 1.452 | 0.557 |  | --- | --- |  | --- | --- |  | --- | --- |
| Constant | 79.378 | <0.001 |  | 79.858 | <0.001 |  | 79.721 | <0.001 |  | 79.721 | <0.001 |
| Akaike information criterion of the model | 1516.910 | --- |  | 1515.253 | --- |  | 1513.606 | --- |  | 1513.606 | --- |
| Likelihood ratio test p-value at this step | --- | 0.575 |  | --- | 0.558 |  | --- | 0.553 |  | --- | 0.553 |

A multivariable linear regression model with a stepwise backward elimination procedure was adopted in order to identify those explanatory variables, which most significantly influence the study participants’ quality of life scores. The data on sociodemographic factors, parasitology, and quality of life of the 187 study participants were collected in the Taabo health demographic surveillance system, south-central Côte d’Ivoire, in June 2010.

^a^ Reference category: male.

^b^ Continuous variable.

^c^ Reference category: no education.

^d^ Reference category: primary sector.

^e^ Reference category: no *S. haematobium* infection.

^f^ Reference category: no *S. mansoni* infection.

^g^ Reference category: no hookworm infection.

^h^ Reference category: no *T. trichiura* infection.

^i^ Reference category: no *Plasmodium* spp. infection.
